# Aging, Cutaneous Burn Injury and Multi-Organ Complications: The Role of the Gut

**DOI:** 10.20900/agmr20220004

**Published:** 2022-06-28

**Authors:** Rachel H. McMahan, Devin M. Boe, Travis M. Walrath, Juan-Pablo Idrovo, Elizabeth J. Kovacs

**Affiliations:** Department of Surgery, Division of GI, Trauma and Endocrine Surgery, and Alcohol Research Program, University of Colorado Denver, Anschutz Medical Campus, Aurora, CO 80045, USA

**Keywords:** advanced age, trauma, intestine, bacteria

## Abstract

Advanced age escalates post-burn complications and older burn patients, and even those with relatively minor burns, have worse clinical outcomes after injury. While the mechanism(s) responsible for the compounding effects of age and burn injury have not been defined, in this viewpoint, we highlight the emerging data suggesting that age-mediated impairment of gut barrier integrity and dysbiosis of the fecal microbiome in older subjects may play a role in the heightened multi-organ responses seen in older patients. Studies aimed at exploring the contribution of intestinal dysfunction in age-related exacerbations of post-burn inflammatory responses could highlight novel therapeutic interventions that can be used to treat victims of burns and other traumatic injuries.

## BURN INJURY IN THE AGED POPULATION

In the US, nearly one million people per year suffer burn injuries, and 40,000 of these patients require hospitalization [1]. Although hospitalization for burn injuries represents ~1% of traumatic injuries, treatment costs exceed $10.4 billion annually [1]. Major contributors to later mortality after burn injury include sepsis, acute respiratory distress syndrome (ARDS) and multiple organ failure (MOF) [1-4]. The most frequent organ to fail after burn injury is the lung, even in the absence of smoke inhalation injury. The lung, by virtue of its massive vascular bed, is exposed to all blood returning to the heart, and is thus an organ that is particularly susceptible to damage following remote injury, including burn. Like other remote injuries, burns affect the lung by triggering a diffuse inflammatory cell infiltrate and the accumulation of extracellular fluid [5,6]. While improvements in resuscitation strategies and wound care have markedly reduced early mortality, clinical evidence demonstrates that advanced age remains a significant risk factor for morbidity after burn and outcomes for patients over the age 65 are very poor [7-12]. As with other major injuries, which result in systemic inflammation, development of pulmonary complications, including pneumonia, ARDS and MOF, contribute to the increased mortality seen in elderly burn patients [13-15]. In addition to increased medical costs and resource utilization, burn injury in advanced age also contributes to lost workdays and long-term disability. As the proportion of the population that is elderly continues to rise, this issue will translate into a greater clinical and socioeconomic burden. Thus, it is imperative that new treatment strategies are developed to combat the effects of age on the response to burn and other forms of traumatic injury.

## THE GUT AS A DRIVER OF SYSTEMIC INFLAMMATION AFTER BURN INJURY

**Figure 1.**
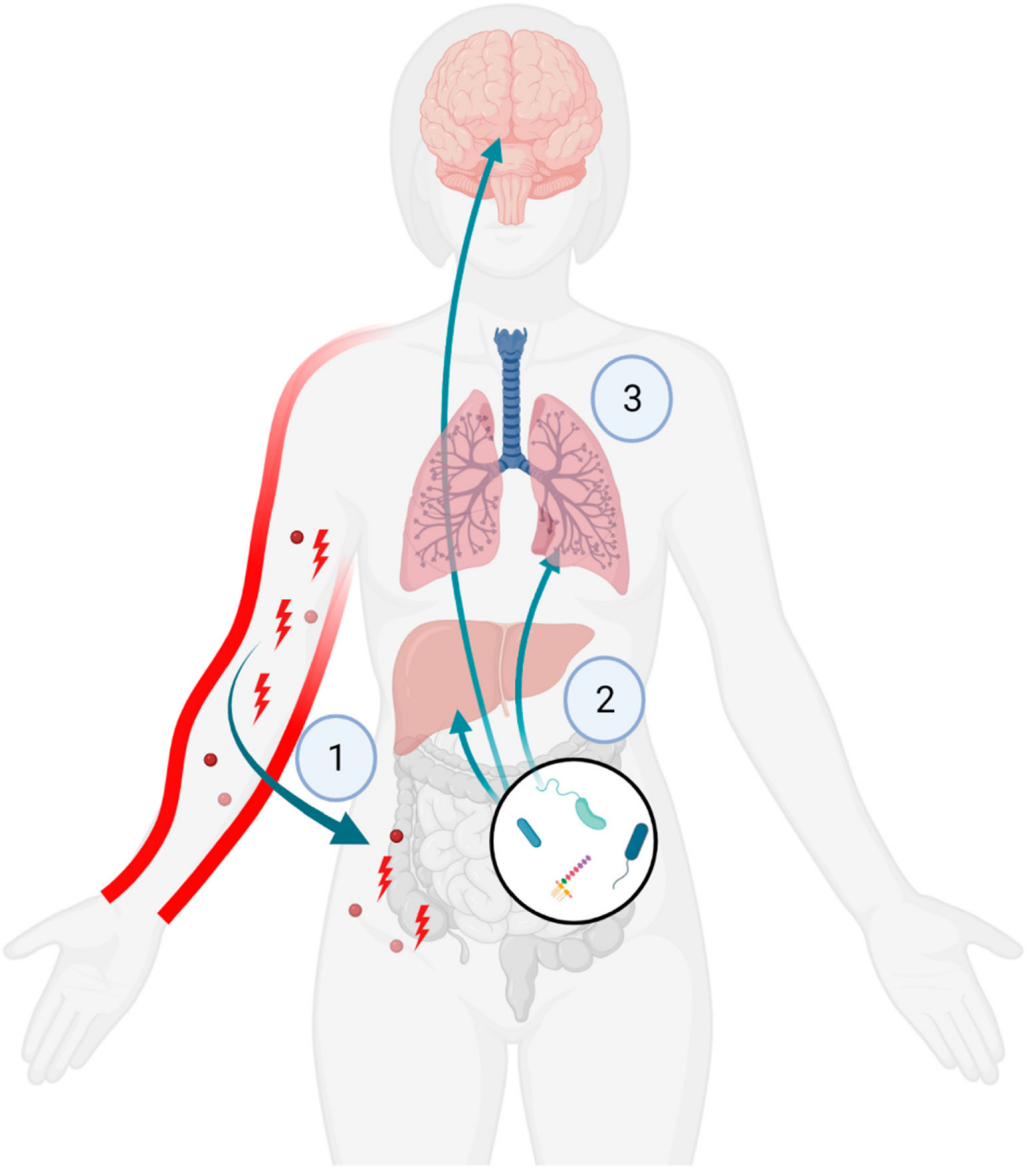
Burn injury and multi-organ inflammation. (**1**) Cytokines, chemokines and DAMPs emanate from burned skin causing (**2**) intestinal damage and release of bacteria and bacterial products into the circulation, resulting in (**3**) heightened systemic inflammation and multi-organ injury.

It is well established that the intestine plays an important role in health and well-being [16,17], as well as critical illness [18]. Aberrant intestinal permeability contributes to heightened systemic inflammation, which can influence immune responses in multiple organs. Thus, the gut is thought to be a driver of systemic inflammation in critical illness [19]. Increasing evidence also demonstrates that intestinal barrier dysfunction contributes to the systematic response to burn injury, mediating clinical outcomes in burn patients. After burn, cytokines, chemokines and danger-associated molecular patterns (DAMPs) emanate from the injured tissue and travel to a number of organs, including the gut [20]. This, combined with post-burn induced intestinal ischemia [21], causes intestinal damage and a breach of the intestinal epithelial barrier, allowing bacteria and their products to enter the lymphatic system and ultimately the blood [22-25]. A breakdown of the gut barrier results in inflammation and damage to distant organs, including the liver, lung and brain (Figure 1). In one study, an 8-fold increase in intestinal permeability was noted within the first day post burn [24]. In others, the extent of gut damage paralleled the magnitude of burn injury in patients in a rodent model [26,27]. Similarly, we and others reported intestinal barrier dysfunction within hours of burn injury in young mice [28-31]. Importantly, repair of gut intestinal integrity by gavage with the intestinal barrier enzyme, intestinal alkaline phosphatase (IAP), improved both systemic inflammation in distal organs along with survival in a murine model of burn injury [20].

The gut microbiota is an important regulator of intestinal barrier function and microbial dysbiosis has been implicated in the response to critical illness, including burn injury [18]. In the healthy gut, the microbiome is composed of bacteria primarily from the Firmicutes, Bacteroidetes, Acitonobacteria and Verrucomicrobia phyla [32]. These bacteria, and others, contribute to barrier integrity, in part, by production of short chain fatty acids (SCFA), which can both enhance mucus production [33] and promote tight junction formation in intestinal epithelial cells [34]. In addition, SCFA induce an anti-inflammatory, tolerogenic immune phenotype of immune cells in the gut, which is critical for intestinal homeostasis [35]. Clinical and animal studies examining dysbiosis of the microbiome reveal a dramatic shift in the fecal microbioime after burn injury, which likely contribute to barrier dysfunction [36-39]. These changes include a burn-induced decrease in overall diversity of bacterial taxa including a reduction in beneficial bacteria such as *Bacteroidaceae*, *Bifidobacterium* and *Ruminococcaceae*. In contrast, levels of the potentially pathogenic bacterial family *Enterobacteriaceae* are increased after burn. While the effects of burn-induced microbial dysbiosis are still being elucidated, a number of species that are decreased after burn are important producers of SCFA and studies in both mice and humans have shown decreased levels of these beneficial metabolites after burn [38,40]. Importantly, in a mouse model of burn injury, fecal microbiome transplant with cecal contents isolated from control mice reversed burn-induced intestinal barrier dysfunction [40].

## POOR OUTCOMES AFTER BURN INJURY IN THE AGED POPULATION: DOES THE GUT PLAY A ROLE?

Little is known about mechanisms driving age-specific exacerbation of burn injury but emerging data would suggest that an exacerbation of burn-induced effects on the gut are likely contributing to this phenotype. In a murine model of age and scald burn injury, our group found that gut barrier dysfunction, as measured by leakage of orally garaged FITC-dextran into the bloodstream, was elevated 8-fold from that of young burned mice [41]. In addition, bacterial burden in the mesenteric lymph nodes was heightened in aged mice compared to young mice 24 h after injury demonstrating that the aged gut is more susceptible to the detrimental effects of burn injury [41]. While the reasons for this heightened response remain to be elucidated, aging has been linked with a number of potentially detrimental physiological changes in the intestine that could be contributing to this dysfunction and warrant further study.

One possible contributing factor could be altered inflammatory responses in age. Trauma, including burns, can cause damage to intestinal epithelial cells (IEC) and disrupt the junctional complexes physically connecting IEC, including tight junctions maintained by proteins zonula occludens protein-1 (ZO-1) and occludin [16,42]. After injury, this connection can be disrupted by the activation of the long form of myosin light-chain kinase (MLCK) [16] by pro-inflammatory cytokines IL-1β, TNFα and IL-6, which are elevated in the blood and tissues after burn [22,43-49]. Advanced age is associated with increased baseline levels of pro-inflammatory cytokines, a phenomenon termed inflamm-aging [50], which could contribute to heighted inflammation after burn. Indeed, it has been observed that a number of pro-inflammatory cytokines and chemokines are elevated to a greater extent in aged burn patients and in murine models of aging and burn injury suggesting the aged population could be more susceptible to inflammatory cytokine-induced intestinal barrier dysfunction after burn [51-56].

In addition to age-related changes in inflammation, the intestine undergoes physiological changes with age which could contribute to the exacerbated post-burn breakdown in the intestinal barrier. Reduced mucosal layer thickness and decreased numbers of mucin producing goblet cells is observed in the ileum and colon of aged mice [57,58]. This mucosal layer is critical for maintaining a physical barrier between IEC and the microbes in the intestine and a loss of this layer leads to increased intestinal inflammation and a breakdown of the epithelial barrier in a number of gastrointestinal diseases [59]. Likewise, age related dysregulation of antimicrobial peptides (AMPs) could also contribute to the altered intestinal response to burn. AMPs, produced by intestinal enterocytes and Paneth cells, reside in the mucosal layer of the gut providing further protection from microbial invasion of IEC via their bactericidal properties [60]. Interestingly, in the context of age, baseline AMP production appears to be moderately increased in the murine intestine, possibly as a compensatory mechanisms for increased baseline levels of inflammation [61-63]. We have reported that in the context of burn-injury, AMP expression is significantly upregulated in the intestines of young mice, most likely to maintain intestinal homeostasis in response to burn-induced microbial dysbiosis and inflammation [62]. However, burn injury in aged mice led instead to an overall reduction in AMP expression to levels equivalent to that of young sham mice, indicating that aged mice are unable to mount an appropriate AMP response in the gut [62].

Finally, changes in the intestinal microbiome in the aged population could also be a factor in the heighted intestinal response to burn injury with age. Evidence in elderly humans and in rodent models of aging show that, even in the absence of injury, there are dramatic changes in fecal microbiota relative to younger subjects (reviewed in [64-68]. In humans, aging often correlates with a decline in bacterial diversity, especially in the context of frailty and unhealthy aging [69-72]. In addition, unhealthy aging is associated with microbial alterations similar to those observed in burn injury, including a loss of beneficial, SCFA producing bacteria such as *Bifidobacterium* and *Ruminococcaceae* [73-75] and increased colonization with opportunistic pathogens such as *Enterbacteriaceae* [76,77]. This age-related dysbiosis could make the aged gut more suceptable to a burn-induced reductions in SCFA, while skewing the microbial population towards a more pro-inflammatory state. Interestingly, our group has recently shown that there is significatly greater dysbiosis of fecal microbiota in aged relative to young burn-injured mice [62] and burn injury in patients over the age of 50 results in age-specific alterations bacterial species diversity and a decrease in beneficial bacteria, such as *Lactobacillus* [78].

## FUTURE DIRECTIONS AND POTENTIAL BIOMARKERS FOR IDENTIFYING AGE-RELATED GUT BARRIER DYSFUNCTION IN BURN INJURY

Although the mechanism responsible for the compounding effects of age and burn injury are likely multifactorial, age-related impairment of gut barrier integrity and dysbiosis of the fecal microbiome in older subjects likely contributes to the heightened multi-organ responses seen in elderly patients. Future studies aimed at reducing post-burn intestinal barrier dysfunction in aged mice to determine if restoration of intestinal function diminishes the increased pulmonary inflammation and mortality is warranted. In addition, identification of a panel of biomarkers of intestinal barrier dysfunction may be able to predict a poor prognosis in elderly burn patients and aid in identifying the subset of patients who require more aggressive supportive care. Intestinal fatty acid binding protein (iFABP) and zonulin are blood-borne biomarkers of intestinal epithelial cell damage and lipopolysaccharide (LPS), and calprotectin are markers of microbial translocation and systemic inflammation [79], respectively. These biomarkers have been found in the blood and/or feces of patients and animal models in critical care conditions that include infection and sepsis [80-88]. For example, iFABP is elevated in the plasma of >90% of adult trauma patients [81]. iFABP also serves as a marker of intestinal damage in early phases of sepsis in critically ill patients [82]. Lastly, heightened levels of plasma iFABP correlated positively with interleukin-6 (IL-6) levels [81], suggesting that there may be a link between gut dysfunction and IL-6 levels. While much remains to be explored, expanding our current knowledge regarding the role of the gut in age-related exacerbations of post-burn inflammatory responses and organ injury has the potential to provide new strategies for developing therapeutic interventions to treat elderly victims of burn and other forms of traumatic injury.
